# A heavy legacy: offspring of malaria-infected mosquitoes show reduced disease resistance

**DOI:** 10.1186/1475-2875-13-442

**Published:** 2014-11-20

**Authors:** Amélie Vantaux, Kounbobr Roch Dabiré, Anna Cohuet, Thierry Lefèvre

**Affiliations:** UMR MIVEGEC (IRD 224 - CNRS 5290 - UM1 - UM2), 911 Avenue Agropolis, BP 64501, 34394 Montpellier Cedex 5, France; IRSS, 01 BP 171, Bobo Dioulasso, Burkina Faso; Centre Muraz, Bobo Dioulasso, Burkina Faso

**Keywords:** *Anopheles coluzzii*, *Anopheles gambiae* M form, *Plasmodium falciparum*, Maternal effects, Malaria

## Abstract

**Background:**

Trans-generational effects of immune stimulation may have either adaptive (trans-generational immune priming) or non-adaptive (fitness costs) effects on offspring ability to fight pathogens.

**Methods:**

*Anopheles coluzzii* and its natural malaria parasite *Plasmodium falciparum* were used to test how maternal parasite infection affected offspring resistance to the same parasite species.

**Results:**

Daughters of exposed mothers had similar qualitative resistance, as measured by their ability to prevent infection, relative to those of control mothers. However, maternal disease exposure altered offspring quantitative resistance, measured as the ability to limit parasite development, with mosquitoes of infected mothers suffering slightly increased parasite intensity compared to controls. In addition, quantitative resistance was minimal in offspring of highly infected mothers, and in offspring issued from eggs produced during the early infection phase.

**Conclusions:**

*Plasmodium falciparum* infection in *An. coluzzii* can have trans-generational costs, lowering quantitative resistance in offspring of infected mothers. Malaria-exposed mosquitoes might heavily invest in immune defences and thereby produce lower quality offspring that are poorly resistant.

**Electronic supplementary material:**

The online version of this article (doi:10.1186/1475-2875-13-442) contains supplementary material, which is available to authorized users.

## Background

Maternal effects, the influence of maternal environment on offspring phenotype, can be an important determinant of host susceptibility to infectious diseases. For example, offspring resistance to parasites can be influenced by maternal temperature [1], food quantity [2], population density [3], and immune stimulation [4].

Trans-generational effects of immune stimulation may have either adaptive or non-adaptive effects on offspring ability to fight pathogens. First, maternal immune activation may be associated with enhanced offspring resistance through the transfer of a memory-like immune response. The existence of such trans-generational immune priming is now well described in both vertebrates and invertebrates [4–6]. On the other hand, immune activation is costly and trade-offs with reproduction and offspring performance are expected [7]. Life history theory posits that organisms dispose of a limited energetic budget, and that any increase in one function can divert resources from other functions [8, 9]. For example, upregulation of the immune system can leave fewer resources for investment in other life-history functions, including competition [10] or reproduction [11]. Such costs at the individual level can extend to subsequent generations. In particular, exposure to pathogens can trigger immune response and decrease investment into reproduction (e.g., smaller eggs) with negative effects on the fitness of future offspring. There is good empirical evidence for such trans-generational costs of maternal immune activation from studies on both vertebrates [12, 13] and invertebrates [14–16]. In these studies, fitness-related traits as varied as body mass, growth, survival, longevity, or fecundity were reduced in offspring from challenged mothers. However, there is little evidence as to whether maternal immune stimulation reduces offspring parasite resistance. Research on bumblebees has indicated that while maternal exposure to heat-kill bacteria increased offspring antibacterial immunity, it decreased their resistance to a trypanosome parasite [17]. It is currently unknown whether maternal exposure to a given parasite species can reduce offspring resistance to the same parasite species.

Mosquitoes are vectors of many deadly diseases, such as malaria, West Nile fever or dengue. Within-generational immune priming has been relatively well described in these medically important insects [18–20] but the existence of maternal effects of immune activation on offspring immunocompetence remains controversial. While a study found no effect of maternal bead inoculation on *Aedes aegypti* melanization response [21], another study showed that offspring of microsporidian-infected *Anopheles gambiae* displayed increased resistance to *Plasmodium berghei* [22]. There is a great diversity of ways in which environmental factors such as maternal infection history can interfere with mosquito competence to malaria parasites [20]. Because any potential increase or decrease in mosquito susceptibility can affect malaria transmission, understanding the impact of maternal infection status on the outcome of mosquito-*Plasmodium* interactions will help making more accurate predictions about the epidemiology of malaria. Here *Anopheles coluzzii* (formerly *An. gambiae* M molecular form [23]), a major vector of human malaria in Africa, was used to examine the effect of maternal exposure to *Plasmodium falciparum* on offspring resistance to the same parasite species.

Most studies on maternal effects on immunity and resistance have focused on offspring from the same reproductive event. Since trans-generational effects can have important epidemiological consequences [24], it is important to gauge the time-scale over which these effects act, not only in a given individual (see e.g. [6]) but also among individuals from successive reproductive cycles. In mosquitoes, blood meals are required to initiate both oogenesis and infection, and egg production therefore coincides with the deployment of immune responses following an infectious meal [19]. This situation is particularly favourable to the occurrence of a trade-off between immune defence and reproduction, and accordingly research in this system shows that mounting an immune response decreased egg production [25].

Here, in addition to comparing *P. falciparum* infection in daughters from malaria-exposed and uninfected females, resistance was quantified in daughters issued from different egg batches produced during two phases of the mothers’ immune response and infection – the early steps of parasite development and the growth of oocysts on the mosquito midgut.

## Methods

Three- to five-day-old female *An. coluzzii* mosquitoes were sourced from an outbred colony established in 2008 from wild-caught females collected in Kou Valley, Burkina Faso. Mosquitoes were maintained under standard insectary conditions (27 ± 2°C, 70 ± 5% relative humidity, 12:12 LD). Experimental infections were performed as described in Sangare et al. [26]. Briefly, females were fed through membranes on gametocyte-infected blood from malaria patients in Burkina Faso, hereafter named infectious (I) blood. Gametocyte densities in blood meals are reported in Additional file 1 and Additional file 2. Venous blood was collected and the volunteer serum was replaced by a non-immune AB serum to avoid human transmission of blocking factors. Control mosquitoes were fed on the same blood in which gametocytes were heat-inactivated, hereafter referred to non-infectious (NI) [27]. This was done to avoid the potential confounding effects of different blood origins on performance of infected and control mosquitoes [27].

Ethical approval was obtained from the Centre Muraz Institutional Ethics Committee (A003-2012/CE-CM). The protocol conforms to the declaration of Helsinki on ethical principles for medical research involving human subjects (version 2002) and informed written consent were obtained from all volunteers.

**Figure 1:**
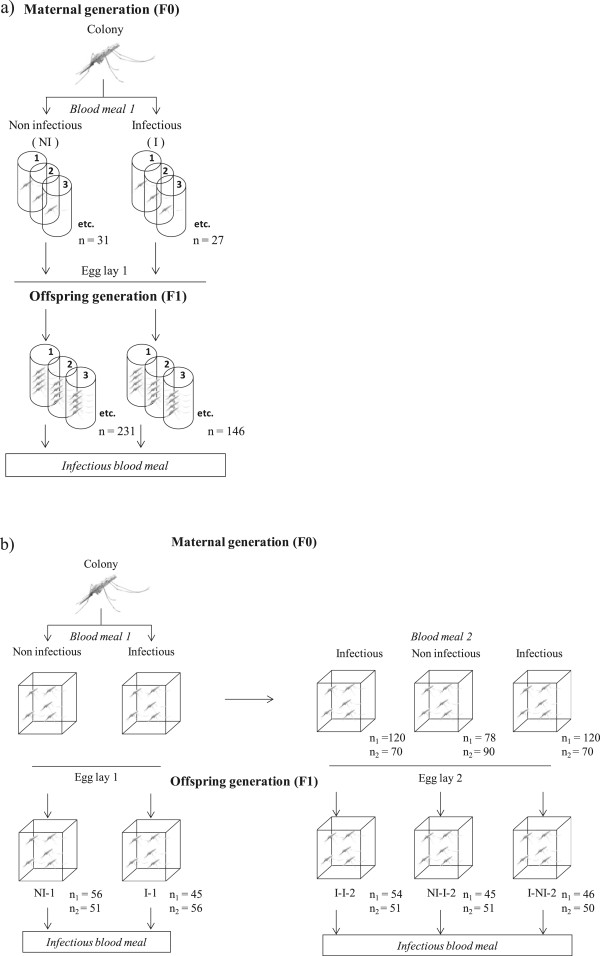
**Experimental design of a) experiment 1 and b) experiment 2.**

Two experiments were carried out (Figure 1). In the first experiment, F0 females received one blood meal (either I or NI) and were then placed in individual cups. Two days afterwards an oviposition site (wet filter paper) was added at the cup bottoms. On day 3 post-blood meal, eggs were retrieved from the cups and transferred to plastic cups filled with water. Larvae were fed *ad libitum* Tetramin^®^ food. Upon emergence, F1 adult mosquitoes belonging to the same brood were transferred to paper cups and provided with 2.5% glucose solution. Three- to five-day-old offspring females of both maternal groups (I and NI) that developed at the same rate (i.e., same age structure) received an infectious blood meal from the same gametocyte carrier on the same day. Fully fed females then returned to their assigned cups. They were dissected eight days later to estimate their resistance to *P. falciparum*. Specifically, infection rate and intensity were quantified. Infection rate is the proportion of infected females, and relates to the mosquito’s ability to prevent infection (qualitative resistance). Infection intensity is the number of oocysts found in the gut of infected females and relates to their ability to limit parasite development (quantitative resistance). These traits were also measured in F0 females (Additional file 1). This experiment used a total of 377 F1 females (from 27 infected and 31 control mothers).

The second experiment was conducted to explore whether trans-generational effects can persist over two gonotrophic cycles. Three days following a first blood meal, F0 females from the infected group received either a second infectious (I-I) or a non-infectious meal (I-NI), while females from the uninfected group received a second infectious blood meal (NI-I) (Figure 1b). Re-feeding propensity was not affected by the nature (I or NI) of the first blood meal (proportion of fed females: 66% in I-I group, 66.5% in I-NI group and 65% in NI-I group; Chi square test, χ_2_^2^ = 0.0094, P = 0.99). Egg batches from each group and each egg lay were retrieved, such that five groups of F1 mosquitoes: I-1, NI-1, I-I-2, I-NI-2, NI-I-2 (Figure 1b) were obtained. Unlike experiment 1 for which F0 mosquitoes were individually tracked, F0 and F1 females were kept in 30 × 30 × 30 cm cages throughout the experiment (Figure 1). This was done because individual mosquito feeding is extremely difficult using membrane assays (low feeding rate, the need for a high number of feeders, high quantity of blood to be drawn to fill each feeders), hence making individual tracking very difficult when mosquitoes are fed more than once. Petri dishes were offered as oviposition sites and the progeny were reared in plastic trays corresponding to their maternal treatment with *ad libitum* Tetramin^®^ food. This experiment was performed twice using a total of 500 F1 females. Offspring females of the different groups developed at similar rate and each group in each egg-lay was fed on the same gametocyte carriers at the same time. Four different gametocyte carriers were used (two for each egg-lay, Figure 1b). Additional file 1 shows infection rate and intensity in F0 females.

Binomial and negative binomial generalized linear mixed models on offspring infection rate and intensity, respectively, were fitted. The best model (Generalized Linear Mixed Model with maternal parasite exposure *vs* GLMM without maternal exposure) was selected based on the Akaike Information Criterion (AIC). Maternal exposure was coded as a fixed factor. Maternal identity (experiment 1) and replicate (experiment 2) were coded as random factors. For experiment 2, the data were also analysed with replicate set as a fixed effect (Additional file 3). The relationship between infection intensity in daughters and mothers was investigated using a negative binomial GLMM. In this analysis, the quadratic term of mother’s infection intensity was included to test for a nonlinear relationship. Finally, the influence of individual body size on infection rate and intensity in the first experiment was explored by including mosquito wing size (a good proxy of body size) in the models. All analyses were performed in R v.3.0.1 using the ‘lme4’ and ‘glmmADMB’ packages.

## Results

In both experiments, maternal parasite exposure did not modify infection rate among offspring issued from the first batch of eggs (Additional file 2 and Additional file 4). Similarly, among second-batch offspring of mothers that received either one or two infectious blood meals, maternal exposure did not contribute to variation in qualitative resistance (Additional file 2 and Additional file 4). In experiment 1, mosquito wing size was not associated with qualitative resistance (ΔAIC = 2, ΔAICc = 2, Additional file 4).

**Figure 2:**
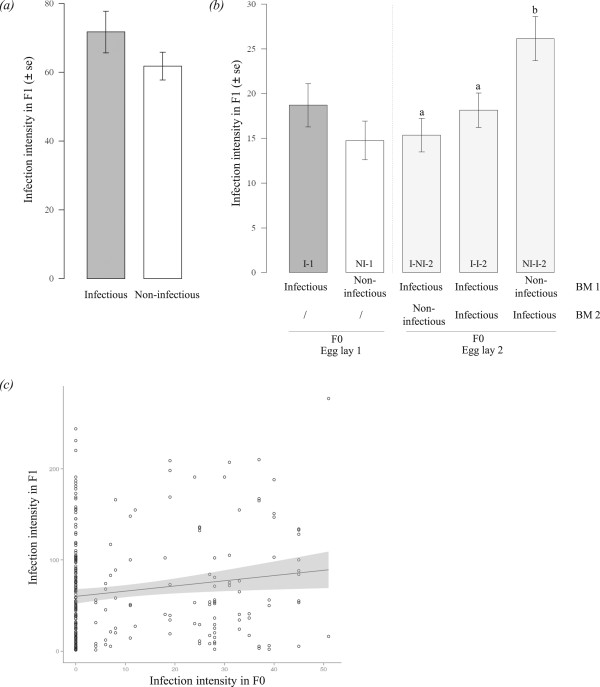
**Trans-generational effects of infection on offspring quantitative resistance (a) in experiment 1; (b) in experiment 2; (c) Positive relationship between infection intensity in F1 and F0 mosquitoes.** The grey area represents the 95% confidence interval. The quadratic term of mother’s infection intensity was not a good predictor (Additional file 6). BM: blood meal, I: infectious, NI: non-infectious. Bars with different letters are significantly different (*post-hoc* Bonferroni-corrected comparisons).

Maternal exposure was marginally related to quantitative resistance in experiment 1 (72 ± 6 oocysts vs 62 ± 4, ΔAIC = 1.6, ΔAICc = 1.7, Figure 2a, Additional file 4), while there was a positive relationship between mosquito size and quantitative resistance (ΔAIC = 4.6, ΔAICc = 4.5). However, neither maternal exposure nor maternal body size influenced offspring size (ΔAIC = 3.3 and 3.8, respectively, ΔAICc = 3.4 and 3.9 respectively, Additional file 5). In experiment 2, maternal exposure was also marginally associated with quantitative resistance with first-batch offspring of exposed mothers harbouring slightly more parasites than those of control mothers (19 ± 2 in I-1 *vs* 15 ± 2 in NI-1, ΔAIC = 1.4, ΔAICc = 1.2, Figure 2b, Additional file 4). However, when first-batch offspring of experiment one and two were pooled together, maternal parasite exposure significantly influenced offspring quantitative resistance (52 ± 4 *vs* 49 ± 3: ΔAIC = 2.3, ΔAICc = 2.3, Additional file 4). In addition, infection intensity in F0 mosquitoes was associated with intensity in offspring (ΔAIC = 2.1, ΔAICc = 2; Figure 2c, Additional file 6). Among second-batch offspring, the best model explaining variation in oocyst number also included maternal treatment (ΔAIC = 3, ΔAICc = 2.8, Figure 2b, Additional file 4). Infection intensity ranged from 15 oocysts ±2 in I-NI-2, to 18 ± 2 in I-I-2 and 26 ± 2 in NI-I-2 offspring. Finally, data from experiment 2, altogether (replicate 1 and 2, egg-lay 1 and 2), indicated that recent maternal exposure to *P. falciparum* strongly affected offspring quantitative resistance (ΔAIC = 5.6, ΔAICc = 5.4, Additional file 7) such that highest intensities were observed in offspring arising from eggs whose development was initiated by an infectious blood meal (i.e., I-1, I-I-2, NI-I-2, Figure 2b).

## Discussion

Parasite intensity was increased in offspring of mothers exposed to *P. falciparum*, a naturally occurring malaria parasite, suggestive that there could be trans-generational costs to immunity in this system. The precise mechanism behind this effect is not yet clear but interactions among host resources, immune responses and parental investment are suspected. *Anopheles coluzzi* mothers could be subject to a trade-off between immune response and offspring quality such that when they were exposed to the parasite, they allocated more resources to their defences at the cost of producing lower quality offspring that were less resistant. In mosquitoes, previous studies have demonstrated that immune function can trade-off with reproduction [25] and that poor general condition can reduce immunocompetence and resistance to *Plasmodium* [28, 29], but see [30]. In other words, stimulating the maternal immune system may compromise offspring quality in this system. Consistent with this idea, a decreased fecundity in offspring of exposed mothers was observed (Additional file 8), even when controlling for the effect of infection intensity in offspring, as previously found in malaria-infected mosquitoes [31–34]. Although further studies are required to investigate the underlying mechanisms, this study is the first to suggest trans-generational costs of infection on offspring resistance to the same parasite species.

Infection intensity was greatest in offspring issued from eggs that developed as a result of an infectious blood meal (Figure 2b: I-1, I-I-2, NI-I-2). This suggests that maternal immune response was maximal and/or most costly during the early steps of parasite development, and accordingly this coincides with the major bottleneck in parasite number [35]. Finally, there was a positive relationship between parasite number in mothers and offspring. While this result confirms that resistance to *P. falciparum* is influenced by mosquito genetics [36], it also supports the idea of trans-generational costs of immunity: the greater the parasite intensity, the greater the maternal immune system was stimulated, and the poorer offspring resistance.

Uninfected control mothers were fed with blood in which parasite infective stages were heat-inactivated [27]. The possibility that the presence of dead gametocytes in the control blood had triggered mosquito immune response cannot be ruled out (see [37] and references therein). Offspring of control mosquitoes would then incur part of the costs resulting from their mothers’ immune response. This would lessen the difference in resistance between offspring from control and infected mothers and it is, therefore, possible that the trans-generational cost of *P. falciparum* exposure on resistance was underestimated here.

Trans-generational defence has been demonstrated in a number of invertebrates [5, 6, 15], but these findings, together with previous studies [14, 21, 38] and refs therein], suggest that it may not be a general phenomenon. These results support the idea that the evolution of trans-generational defence depends on host and parasite life history [6, 21, 37]. Unlike bumblebees [6] or mealworm beetle [15], the risk of mosquito contact with malaria parasites is not influenced by maternal infection status, suggesting that there would be limited benefits of trans-generational immune priming in this system.

## Conclusions

Quantitative resistance was slightly lower in offspring of malaria-exposed mothers. The extent to which this small reduction in resistance will contribute to disease dynamic is currently unclear but deserves consideration. Future studies combining the effects of environmental stressors, such as food limitation [37] with maternal infection history, are required to better assess the extent of maternally transmitted fitness costs on offspring resistance to *P. falciparum* in natural conditions. Finally, this work not only highlights the importance of maternal parasite exposure in understanding the outcome of mosquito-malaria interactions, it also contributes to the current understanding of the non-genetic inheritance of host resistance in natural populations [38].

## Electronic supplementary material

Additional file 1: **Infection rate and intensity in F0 females.** The data provided represent the infection rate (±95% CI) and intensity (± se) in F0 females. (DOCX 13 KB)

Additional file 2: **Infection rate and intensity in F1 females.** The data provided represent the infection rate (±95% CI) and intensity (± se) in F0 females. (DOCX 14 KB)

Additional file 3: **Term significance following statistical analyses of experiment 2 treating replicate as a fixed factor and using Likelihood Ratio Tests.** The data provided represent the statistical analyses ran on experiment 2 treating replicate as a fixed factor and using likelihood ratio test. (DOCX 14 KB)

Additional file 4: **Selection of models fitted on infection rate (qualitative resistance) or infection intensity (quantitative resistance) using Akaike’s information Criteria (AIC).** The data provided represent the statistical analyses used on models selection on infection rate and infection intensity. (DOCX 16 KB)

Additional file 5: **Selection of models fitted on offspring wingsize using Akaike’s information Criteria (AIC).** The data provided represent the statistical analyses used on models selection on offspring wingsize. (DOCX 12 KB)

Additional file 6: **Selection of models fitted on infection intensity (quantitative resistance) using Akaike’s information Criteria (AIC) including maternal intensity and its quadratic term.** The data provided represent the statistical analyses used on models selection to test the correlation between maternal and offspring intensity. (DOCX 12 KB)

Additional file 7: **Selection of models fitted on infection intensity (quantitative resistance) using Akaike’s information Criteria (AIC) including last infectious blood-meal variable and gametocytemia.** The data provided represent the statistical analyses used on models selection to test the effect of the last infectious blood meal and gametocytemia in experiment 2. (DOCX 13 KB)

Additional file 8: **Proportion of gravid females in offspring of exposed and unexposed mothers from experiment 1 and 2.** The data provided represent the figure of the proportion of gravid females offspring and the corresponding statistical analyses. (DOCX 42 KB)

## Abbreviations

AIC: Akaike information criterion (AIC)
LD: Light–dark
GLMM: Generalized linear mixed models.
