# Supplementary material for: A heavy legacy: offspring of malaria-infected mosquitoes show reduced disease resistance
Source: Malar J. 2014 Nov 20;13:442. doi: 10.1186/1475-2875-13-442 (PMC4255934; doi:10.1186/1475-2875-13-442)
Supplement: Supplementary file 1 — Additional file 1: Infection rate and intensity in F0 females. The data provided represent the infection rate (±95% CI) and intensity (± se) in F0 females. (DOCX 13 KB) [file 12936_2014_3611_MOESM1_ESM.docx]

**Additional file 1: Table S1: Infection rate and intensity in F0 females**. I = mothers that received a single infectious blood-meal, NI = mothers that received a single heat inactivated non-infectious blood-meal (note that none of the NI females were infected), I-I = mothers that received two successive infectious blood-meal, I-NI = mothers that received a first infectious blood-meal and a second non-infectious blood-meal, NI-I = mothers that received a first non-infectious blood-meal and a second infectious blood-meal. Gametocyte density: number of gametocytes per 1000 leucocytes.

| **Experiment** |  | **Mosquito** | **Infection rate** | **Infection** | **Gametocyte** |
| --- | --- | --- | --- | --- | --- |
|  |  | **group** | **± 95% CI** | **intensity ± se** | **density** |
| 1 |  | I | 1 ± 0 | 23.34 ± 2.52 | 16 |
|  |  | NI | - | - | - |
| 2 | Replicate 1 | I-I | 0.88 ± 0.05 | 10.9 ± 0.85 | 24 & 14 |
|  |  | I-NI | 0.86 ± 0.07 | 5.1 ± 0.45 | 24 |
|  |  | NI-I | 0.69 ± 0.09 | 12.66 ± 1.42 | 14 |
|  | Replicate 2 | I-I | 0.74 ± 0.1 | 30.98 ± 5.82 | 39 & 23 |
|  |  | I-NI | 0.79 ± 0.1 | 35.94 ± 4.77 | 39 |
|  |  | NI-I | 0.29 ± 0.09 | 10.19 ± 2.56 | 23 |
