# Supplementary material for: A heavy legacy: offspring of malaria-infected mosquitoes show reduced disease resistance
Source: Malar J. 2014 Nov 20;13:442. doi: 10.1186/1475-2875-13-442 (PMC4255934; doi:10.1186/1475-2875-13-442)
Supplement: Supplementary file 2 — Additional file 2: Infection rate and intensity in F1 females. The data provided represent the infection rate (±95% CI) and intensity (± se) in F0 females. (DOCX 14 KB) [file 12936_2014_3611_MOESM2_ESM.docx]

**Additional file 2: Table S2: Infection rate and intensity in F1 females**. I-1 = daughters from I mothers and first batch of eggs, NI-1 = daughters from NI mothers and first batch of eggs, I-I-2 = daughters from I-I mothers and second batch of eggs, I-NI-2 = daughters from I-NI mothers and second batch of eggs, NI-I-2 = daughters from NI-I mothers and second batch of eggs. Gametocyte density: number of gametocytes per 1000 leucocytes.

| **Experiment** |  | **Mosquito** | **Infection rate** | **Infection** | **Gametocyte** |
| --- | --- | --- | --- | --- | --- |
|  |  | **group** | **± 95% CI** | **intensity ± se** | **density** |
| 1 |  | I-1 | 0.71 ± 0.07 | 71.77 ± 6.06 | 47 |
|  |  | NI-1 | 0.75 ± 0.06 | 61.8 ± 4.02 |  |
| 2 | Replicate  1 | I-1 | 0.6 ± 0.14 | 12.96 ± 2.64 | 19 |
|  |  | NI-1 | 0.38 ± 0.13 | 7 ± 2.4 |  |
|  |  | I-I-2 | 0.57 ± 0.13 | 8.55 ± 1.86 | 15 |
|  |  | I-NI-2 | 0.54 ± 0.14 | 14.68 ± 3.05 |  |
|  |  | NI-I-2 | 0.6 ± 0.14 | 9.78 ± 1.78 |  |
|  | Replicate  2 | I-1 | 0.65 ± 0.13 | 23.39 ± 3.65 | 24 |
|  |  | NI-1 | 0.8 ± 0.11 | 18.73 ± 2.84 |  |
|  |  | I-I-2 | 0.9 ± 0.08 | 24.59 ± 2.57 | 7 |
|  |  | I-NI-2 | 0.8 ± 0.11 | 15.78 ± 2.43 |  |
|  |  | NI-I-2 | 0.78 ± 0.11 | 37.17 ± 2.8 |  |
