# Supplementary material for: A heavy legacy: offspring of malaria-infected mosquitoes show reduced disease resistance
Source: Malar J. 2014 Nov 20;13:442. doi: 10.1186/1475-2875-13-442 (PMC4255934; doi:10.1186/1475-2875-13-442)
Supplement: Supplementary file 3 — Additional file 3: Term significance following statistical analyses of experiment 2 treating replicate as a fixed factor and using Likelihood Ratio Tests. The data provided represent the statistical analyses ran on experiment 2 treating replicate as a fixed factor and using likelihood ratio test. (DOCX 14 KB) [file 12936_2014_3611_MOESM3_ESM.docx]

**Additional file 3: Table S7: Term significance following statistical analyses of experiment 2 treating replicate as a fixed factor and using Likelihood Ratio Tests.** ME= maternal exposure, LIBM=last infectious blood meal, G=gametocytemia.

| **Experiment** | **Egg-lay** | **Parameter** | **Competing models** | **df** | **Χ^2^** | **P** |
| --- | --- | --- | --- | --- | --- | --- |
| 2 | 1 | Qualitative  resistance | ME*Replicate | 1 | 8.06 | 0.004 |
|  |  |  | ME | 1 | 0.23 | 0.63 |
|  |  |  | Replicate | 1 | 13.22 | 0.0002 |
|  |  | Quantitative  resistance | ME*Replicate | 1 | 0.76 | 0.39 |
|  |  |  | ME | 1 | 2.8 | 0.095 |
|  |  |  | Replicate | 1 | 12.99 | 0.0003 |
|  | 2 | Qualitative  Resistance | ME*Replicate | 2 | 2.25 | 0.33 |
|  |  |  | ME | 2 | 1.2 | 0.55 |
|  |  |  | Replicate | 1 | 24.33 | >0.0001 |
|  |  | Quantitative  resistance | ME*Replicate | 2 | 16.3 | 0.0003 |
|  |  |  | ME | 2 | 17.7 | 0.0002 |
|  |  |  | Replicate | 1 | 51.7 | >0.0001 |
| 2 | 1&2 | LIBM | LIBM*Replicate | 1 | 5.046 | 0.025 |
|  |  | Quantitative  resistance | LIBM | 1 | 10.76 | 0.001 |
|  |  |  | G | 1 | 0.05 | 0.82 |
|  |  |  | Replicate | 1 | 40.4 | >0.0001 |
|  |  |  | Egg-lay | 1 | 0.02 | 0.9 |
