# Supplementary material for: A heavy legacy: offspring of malaria-infected mosquitoes show reduced disease resistance
Source: Malar J. 2014 Nov 20;13:442. doi: 10.1186/1475-2875-13-442 (PMC4255934; doi:10.1186/1475-2875-13-442)
Supplement: Supplementary file 4 — Additional file 4: Selection of models fitted on infection rate (qualitative resistance) or infection intensity (quantitative resistance) using Akaike’s information Criteria (AIC). The data provided represent the statistical analyses used on models selection on infection rate and infection intensity. (DOCX 16 KB) [file 12936_2014_3611_MOESM4_ESM.docx]

**Additional file 4: Table S3: Selection of models fitted on infection rate (qualitative resistance) or infection intensity (quantitative resistance) using Akaike’s information Criteria (AIC)**. The promoted model by the least Akaike information criterion (AIC) value is highlighted in bold. ME= maternal exposure, WZ = mosquito wing size.

| **Experiment** | **Egg-lay** | **Parameter** | **Competing models** | **ΔAIC** | **ΔAICc** | **AIC-value** | **df** | **AIC-weight** |
| --- | --- | --- | --- | --- | --- | --- | --- | --- |
| 1 | 1 | Qualitative  resistance | Model including ME & WZ | 4 | 4.1 | 328.2 | 4 | 0.07 |
|  |  |  | Model including WZ only | 2 | 2 | 326.2 | 3 | 0.2 |
|  |  |  | Model including ME only | 2 | 2 | 326.2 | 3 | 0.2 |
|  |  |  | **Model without ME & WZ** | **0** | **0** | **324.2** | **2** | **0.54** |
|  |  | Quantitative  resistance | Model including ME & WZ | 1.6 | 1.7 | 2166.7 | 5 | 0.28 |
|  |  |  | **Model including WZ only** | **0** | **0** | **2165.1** | **4** | **0.6** |
|  |  |  | Model including ME only | 4.7 | 4.7 | 2169.8 | 4 | 0.06 |
|  |  |  | Model without ME & WZ | 4.6 | 4.5 | 2169.7 | 3 | 0.06 |
| 2 | 1 | Qualitative | Model including ME | 1.7 | 1.8 | 271.18 | 3 | 0.3 |
|  |  | resistance | **Model without ME** | **0** | **0** | **269.42** | **2** | **0.7** |
|  |  | Quantitative | **Model including ME** | **0** | **0** | **936.05** | **4** | **0.67** |
|  |  | resistance | Model without ME | 1.4 | 1.2 | 937.438 | 3 | 0.33 |
|  | 2 | Qualitative | Model including ME | 2.8 | 2.9 | 350.8 | 4 | 0.19 |
|  |  | Resistance | **Model without ME** | **0** | **0** | **347.97** | **2** | **0.81** |
|  |  | Quantitative | **Model including ME** | **0** | **0** | **1645.28** | **5** | **0.81** |
|  |  | resistance | Model without ME | 3 | 2.8 | 1648.24 | 3 | 0.19 |
| 1+2 | 1 | Qualitative | Model including ME | 1.8 | 1.8 | 713.22 | 3 | 0.29 |
|  |  | Resistance | **Model without ME** | **0** | **0** | **711.4** | **2** | **0.71** |
|  |  | Quantitative | **Model including ME** | **0** | **0** | **3808.52** | **4** | **0.76** |
|  |  | resistance | Model without ME | 2.3 | 2.3 | 3810.84 | 3 | 0.24 |
