# Supplementary material for: A heavy legacy: offspring of malaria-infected mosquitoes show reduced disease resistance
Source: Malar J. 2014 Nov 20;13:442. doi: 10.1186/1475-2875-13-442 (PMC4255934; doi:10.1186/1475-2875-13-442)
Supplement: Supplementary file 5 — Additional file 5: Selection of models fitted on offspring wingsize using Akaike’s information Criteria (AIC). The data provided represent the statistical analyses used on models selection on offspring wingsize. (DOCX 12 KB) [file 12936_2014_3611_MOESM5_ESM.docx]

**Additional file 5: Table S4: Selection of models fitted on offspring wingsize using Akaike’s information Criteria (AIC)**. We compared models with and without Maternal Exposure (ME) maternal wingsize (MWZ). The promoted model by the least Akaike information criterion (AIC) value is highlighted in bold.

| **Experiment** | **Egg-lay** | **Parameter** | **Competing models** | **ΔAIC** | **ΔAICc** | **AIC-value** | **df** | **AIC-weight** |
| --- | --- | --- | --- | --- | --- | --- | --- | --- |
| 1 | 1 | Offspring wingsize | Model including ME & MWZ | 7.1 | 7.2 | 490.99 | 5 | 0.72 |
|  |  |  | Model including MWZ only | 3.8 | 3.9 | 494.3 | 4 | 0.15 |
|  |  |  | Model including ME only | 3.3 | 3.4 | 494.8 | 4 | 0.11 |
|  |  |  | **Model without ME & MWZ** | **0** | **0** | **498.07** | **3** | **0.73** |
