# Supplementary material for: A heavy legacy: offspring of malaria-infected mosquitoes show reduced disease resistance
Source: Malar J. 2014 Nov 20;13:442. doi: 10.1186/1475-2875-13-442 (PMC4255934; doi:10.1186/1475-2875-13-442)
Supplement: Supplementary file 6 — Additional file 6: Selection of models fitted on infection intensity (quantitative resistance) using Akaike’s information Criteria (AIC) including maternal intensity and its quadratic term. The data provided represent the statistical analyses used on models selection to test the correlation between maternal and offspring intensity. (DOCX 12 KB) [file 12936_2014_3611_MOESM6_ESM.docx]

**Additional file 6: Table S5: Selection of models fitted on infection intensity (quantitative resistance) using Akaike’s information Criteria (AIC)**. We compared models with and without Maternal Intensity (MI) and its Quadratic Term (QT). The promoted model by the least Akaike information criterion (AIC) value is highlighted in bold.

| **Experiment** | **Egg-lay** | **Parameter** | **Competing models** | **ΔAIC** | **ΔAICc** | **AIC-value** | **df** | **AIC-weight** |
| --- | --- | --- | --- | --- | --- | --- | --- | --- |
| 1 | 1 | Quantitative  resistance | Model including MI & QT | 1.7 | 1.8 | 2867.52 | 5 | 0.24 |
|  |  |  | **Model including MI only** | **0** | **0** | **2865.82** | **4** | **0.56** |
|  |  |  | Model without MI & QT | 2.1 | 2 | 2867.88 | 3 | 0.2 |
