# Supplementary material for: A heavy legacy: offspring of malaria-infected mosquitoes show reduced disease resistance
Source: Malar J. 2014 Nov 20;13:442. doi: 10.1186/1475-2875-13-442 (PMC4255934; doi:10.1186/1475-2875-13-442)
Supplement: Supplementary file 7 — Additional file 7: Selection of models fitted on infection intensity (quantitative resistance) using Akaike’s information Criteria (AIC) including last infectious blood-meal variable and gametocytemia. The data provided represent the statistical analyses used on models selection to test the effect of the last infectious blood meal and gametocytemia in experiment 2. (DOCX 13 KB) [file 12936_2014_3611_MOESM7_ESM.docx]

**Additional file 7: Table S6: Selection of models fitted on infection intensity (quantitative resistance) using Akaike’s information Criteria (AIC)**. We compared models with and without the binomial variable “Last Infectious Blood-Meal” (yes/no) and including gametocytemia as a covariate to account for the infectious dose received. The promoted model by the least Akaike information criterion (AIC) value is highlighted in bold. LIBM=last infectious blood meal, G=gametocytemia.

| **Experiment** | **Egg-lay** | **Parameter** | **Competing models** | **ΔAIC** | **ΔAICc** | **AIC-value** | **df** | **AIC-weight** |
| --- | --- | --- | --- | --- | --- | --- | --- | --- |
|  |  | Quantitative  Resistance | **Model including LIBM & G** | **0** | **0** | **2576.96** | **6** | **0.604** |
| 2 | 1&2 |  | Model including LIBM only | 1 | 1 | 2578 | 5 | 0.359 |
|  |  |  | Model without LIBM & G | 5.6 | 5.4 | 2582.52 | 4 | 0.037 |
