# Supplementary material for: A heavy legacy: offspring of malaria-infected mosquitoes show reduced disease resistance
Source: Malar J. 2014 Nov 20;13:442. doi: 10.1186/1475-2875-13-442 (PMC4255934; doi:10.1186/1475-2875-13-442)
Supplement: Supplementary file 8 — Additional file 8: Proportion of gravid females in offspring of exposed and unexposed mothers from experiment 1 and 2. The data provided represent the figure of the proportion of gravid females offspring and the corresponding statistical analyses. (DOCX 42 KB) [file 12936_2014_3611_MOESM8_ESM.docx]

**Additional file 8: Figure S1. Proportion of gravid females in offspring of exposed and unexposed mothers from experiment 1 and 2.** Egg incidence was determined 8 days post blood meal during mosquitoes dissection to determine quantitative resistance. Maternal exposure was a strong predictor of egg incidence (ΔAIC=7.6, ΔAICc=7.6). Error bars show 95% confidence limits.
